# Intravitreal bevacizumab plus propranolol for neovascular age-related macular degeneration (the BEVALOL study): a phase I clinical trial

**DOI:** 10.1186/s40942-023-00460-1

**Published:** 2023-04-13

**Authors:** José Edísio da Silva Tavares Neto, Francyne Veiga Reis Cyrino, Moises Moura Lucena, Ingrid U. Scott, André Márcio Vieira Messias, Rodrigo Jorge

**Affiliations:** 1 Department of Ophthalmology, Ribeirão Preto Medical School, University of São Paulo, 3900, Bandeirantes av., Ribeirão Preto, 14048-900 Brazil; 2 Departments of Ophthalmology and Public Health Sciences, Penn State College of Medicine, Hershey, PA USA

**Keywords:** Age-related macular degeneration, VEGF, Propranolol, Bevacizumab, Maculopathy

## Abstract

**Background:**

Given the persistently large public health impact of neovascular age-related macular degeneration (nARMD) despite many years of anti-VEGF therapy as the first-line treatment and the demonstrated ability of b-blockers to reduce neovascularization, a synergistic effect between an anti-VEGF agent and an intravitreal beta-blocker is important to investigate in the quest for therapeutic alternatives that maximize efficacy and/or reduce costs. The main purpose of this study is to investigate the safety of a 0.1 ml intravitreal injection of a combination of bevacizumab (1.25 mg/0.05 ml) and propranolol (50 g/0.05 ml) to treat nARMD.

**Methods:**

Prospective phase I clinical trial that included patients with nARMD. Comprehensive ophthalmic evaluation was performed at baseline and included Early Treatment Diabetic Retinopathy Study (ETDRS) best-corrected visual acuity (BCVA), biomicroscopy of the anterior and posterior segments, binocular indirect ophthalmoscopy, color fundus photography, spectral domain optical coherence tomography (OCT), OCT angiography (OCT-A), fluorescein angiography (Spectralis, Heidelberg), and full-field electroretinography (ERG). All eyes were treated with a 0.1 ml intravitreal injection of a combination of bevacizumab (1.25 mg/0.05 ml) and propranolol (50 g/0.05 ml) within 1 week of baseline evaluation. The patients were reexamined at weeks 4, 8 and 12, and clinical evaluation and SD-OCT were performed at all follow-up visits. Additional injections of combination bevacizumab (1.25 mg/0.05 ml) and propranolol (50 g/0.05 ml) were administered at weeks 4 and 8. At the final study evaluation (week 12), color fundus photography, OCT-A, fluorescein angiography, and full-field ERG were repeated.

**Results:**

Eleven patients (11 eyes) completed all study visits of the 12 week study. Full field ERG b-waves did not show significant (p < 0.05) changes at week 12 compared to baseline. During the 12 week follow-up period, none of the study eyes developed intraocular inflammation, endophthalmitis or intraocular pressure elevation more than 4 mmHg over baseline. Mean ± SE BCVA (logMAR) was 0.79 ± 0.09 at baseline and was significantly (p < 0.05) improved to 0.61 ± 0.10 at week 4; 0.53 ± 0.10 at week 8; and 0.51 ± 0.09 at week 12. Mean ± SE central subfield thickness (CST) (μm) was 462 ± 45 at baseline and was significantly (p < 0.05) lower at 4, 8 and 12 weeks (385 ± 37; 356 ± 29 and 341 ± 24, respectively).

**Conclusions:**

In this 12 week trial of a combination of intravitreal bevacizumab and propranolol for treatment of nARMD, no adverse events or signals of ocular toxicity were observed. Further studies using this combination therapy are warranted.

*Trial Registration* Project registered in Plataforma Brasil with CAAE number 28108920.0.0000.5440 and approved in ethics committee of Clinics Hospital of Ribeirao Preto Medicine School of São Paulo University—Ribeirão Preto, São Paulo, Brazil (appreciation number 3.999.989 gave the approval).

## Background

Neovascular age-related macular degeneration (nARMD) is one of the major causes of visual impairment. In subretinal neovascularization, new vessels may originate from the deep retinal capillary bed and grow through the photoreceptor layer into the subretinal space (retinal angiomatous proliferation), or they may originate from choroidal vessels and extend through Bruch's membrane and the retinal pigment epithelium (RPE) (choroidal neovascularization). Subretinal neovascularization, in either form, is the hallmark of nARMD and has serious consequences regarding vision loss. [1]

The mainstay of treatment for nARMD is the administration of intravitreal anti-vascular endothelial growth factor (anti-VEGF) therapy [2, 3]. However, there are several challenges associated with this treatment. First, intravitreal injections in some patients need to be administered frequently and for long periods of time. Second, anti-VEGF treatment may be associated with systemic thromboembolic events[4, 5] and local adverse events, including RPE tears [6], retinal tears [7], retinal detachment[8], elevation of intraocular pressure (IOP) [9] and endophthalmitis [10]. Third, some patients demonstrate resistance or tachyphylaxis to anti-VEGF monotherapy [11, 12]. Fourth, there is considerable concern regarding the high cost of anti-VEGF drugs [13]. Given these challenges associated with intravitreal anti-VEGF monotherapy, we investigated the use of propranolol as an adjuvant to bevacizumab, both as a means of increasing the efficacy of treatment against the disease, as well as to increase the spacing between doses of antiangiogenic agents.

Propranolol, a nonspecific beta-adrenergic receptor (b-AR) antagonist, has become the gold standard for the treatment of severe childhood hemangioma [14]. In addition, a study reported the use of intravitreal injection of propranolol to treat a retinal capillary hemangioma in a patient with Von Hippel Lindau [15]. The tumor regressive properties of propranolol stem from its ability to inhibit expression of VEGF [16] and, therefore, the b-AR antagonism of propranolol may be useful in the treatment of ocular posterior segment neovascular diseases. [17]

In mice with oxygen-induced ischemic retinopathy (OIR), both propranolol treatment and specific b2-AR blockade inhibit angiogenesis via attenuation of endothelial cell proliferation, migration and differentiation, in addition to inhibiting VEGF overexpression [18, 19]. A study [20] showed that intravitreal propranolol was associated with a reduction in choroidal neovascularization (CNV) area by 50%, and that specific b2-AR blockade decreases VEGF expression in mouse choroidal endothelial cells and RPE cells. Another study [21] extended these findings to human fetal RPE cells in culture.

Retrospective investigations in humans have shown that oral b-blocker treatment is correlated with a reduced number of anti-VEGF injections in patients with nARMD [22]. Further, a prospective single-arm study in patients with persistent retinal fluid despite maximal anti-VEGF therapy for nARMD showed that topical treatment with timolol-dorzolamide, in addition to anti-VEGF therapy, was associated with greater reduction of retinal fluid compared to anti-VEGF monotherapy. [23]

Given the persistently large public health impact of nARMD despite many years of anti-VEGF therapy as the first-line treatment and the aforementioned demonstrated effects of b-blockers, a synergistic effect between an anti-VEGF agent and an intravitreal b-blocker is important to investigate in the quest for therapeutic alternatives that maximize efficacy and/or reduce costs. Towards this end, we conducted a phase I clinical trial to assess the safety of a 0.1 ml intravitreal injection of a combination of bevacizumab (1.25 mg/0.05 ml) and propranolol (50 g/0.05 ml) to treat patients with nARMD.

## Materials and methods

### Study design

This prospective study adhered to the tenets of the Declaration of Helsinki and was approved by the local institutional research ethics committee. Consecutive patients diagnosed with nARMD in the Department of Ophthalmology, Ribeirão Preto Medical School, University of São Paulo between May 2020 and February 2021 were enrolled after written informed consent was obtained. The informed consent included information concerning off-label use of intravitreal bevacizumab and propranolol.

### Study population

Inclusion criteria were: (1) age over 18 years; (2) diagnosis of subretinal neovascular membrane associated with ARMD (nARMD); (3) absence of clinically significant lens opacity, adequate pupillary dilation and sufficient patient cooperation to permit complete ocular examinations. Exclusion criteria were: (1) subfoveal fibrosis; (2) any clinical condition that impairs fundus documentation or patient follow-up; (3) medical or psychological conditions that prevent providing fully informed consent; (4) allergy to propranolol hydrochloride or bevacizumab, or to other drugs used during preparation for intravitreal injections; (5) allergy to the use of intravenous fluorescein dye; (6) pregnancy, breastfeeding or pregnancy plans in the subsequent 6 months.

### Baseline and follow-up evaluations

After determination of study eligibility, comprehensive ophthalmic evaluation was performed at baseline and included logMAR best-corrected visual acuity (BCVA) measured according to the standardized Early Treatment Diabetic Retinopathy Study (ETDRS) protocol using ETDRS charts. Applanation tonometry with a Goldmann tonometer, binocular indirect ophthalmoscopy, color fundus photography, spectral domain optical coherence tomography (OCT), OCT angiography (OCT-A), fluorescein angiography and full-field electroretinography (ERG) (Diagnosys LLC, USA) using International Society for Clinical Electrophysiology of Vision (ISCEV) standard protocols [24] (including dark-adapted: 0.01 cd-s/m2 [ROD], 3.0 cd-s/m2 [COMBINED]/light-adapted [30 cd/m^2^]: 3.0 cd.s/m^2^ [CONE], and 3.0 cd.s/m^2^—30 Hz [FLICKER] responses) were also performed.

OCT examinations were performed using the Spectralis^®^ HRA + OCT image system (Heidelberg Engineering, Germany). The center of the OCT scan was determined at baseline by the center of the fovea based on patient fixation. At subsequent visits, the automatic follow-up function on the Heidelberg machine was used to scan the same macular region as was scanned during the previous visit. The strategy for analysis of central subfield thickness (CST) was based on a grid thickness map generated automatically by the software.

All eyes were treated with an intravitreal injection of 0.10 ml, containing 0.05 ml (1.25 mg) of bevacizumab and 0.05 ml of propranolol (50 g) within 1 week of the baseline evaluation. The patients were followed up with serial ophthalmic examinations including ETDRS BCVA measurement, slit lamp and OCT examinations at 4, 8 and 12 weeks after the injection, with additional bevacizumab and propranolol combined injections administered at 4 and 8 weeks, for a total of 3 injections.

At the final study visit (12 weeks after the initial injection), all the assessments performed at baseline were repeated: BCVA measurement, tonometry, binocular indirect ophthalmoscopy, color fundus photography, OCT, OCT-A, fluorescein angiography and full-field ERG.

### Treatment protocol

Bevacizumab (1.25 mg/0.05 ml) (Avastin^®^; Genentech, South San Francisco, Califórnia, EUA, osmolality 342 mOsm/kg) and propranolol (50 μg/0.05 ml) (Propranolol 1 mg/ml; Citopharma compounding pharmacy, Belo Horizonte, Minas Gerais, Brazil, osmolality 12 mOsm/kg) were administered in combination through the pars plana. Bevacizumab (0.05 ml) was aspirated from its commercial 100 mg-vial and propranolol (0.05 ml) from a compound pharmacy 1 ml-vial. Both drugs were added to a disposable BD Ultra-Fine^™^ 29G ½ inch syringe. The injection was performed under topical anesthesia, 3 mm posterior to the limbus in pseudophakic patients and 3.5 mm posterior to the limbus in phakic patients. Unless medically contraindicated, patients were treated with an oral dose of 250 mg acetazolamide 30 min prior to the injection. After the injection, perfusion of the optic nerve was confirmed by indirect ophthalmoscopy. Anterior chamber paracentesis was performed if ophthalmoscopy indicated impaired optic disc or retinal perfusion. Patients were instructed to use topical moxifloxacin 0.5%, one drop every 6 h, to the study eye, starting three days before the injection and continuing for 1 week after the injection.

### Outcome measures

Safety outcomes assessed include mean a- and b-wave amplitude change on ERG, intraocular pressure (IOP) elevation, change in BCVA, signs of intraocular inflammation (anterior chamber cells or flare), and progression of cataract. Although not a safety outcome, CST was also assessed prospectively.

### Statistical analysis

Data are reported as mean ± standard error (SE). Continuous data (CST, BCVA, IOP) measured at each follow-up visit were compared using a Multiple Analysis of Variance (MANOVA) for repeated measurements. ERG amplitudes and implicit times measured at baseline and follow-up were compared using paired t-test.

## Results

Thirteen eyes from 13 patients were enrolled in the study and two patients were lost to follow-up as they did not attend two consecutive appointments due to personal concerns during COVID-19 pandemics. Demographic characteristics of the patients are summarized in Table 1. Seven (63.6%) of the patients were men and patients’ mean age was 73.54 ± 7.57 years.

**Table 1:** Patients’ demographics, central subfield thickness, best-corrected visual acuity and intraocular pressure at baseline and 12-week study visits

Patient	Information	Baseline	Follow-Up	Sex/age/eye
1	CST (μm)	485	293	M/65/R
	BCVA (LogMAR)	20/80	20/20	
	IOP (mmHg)	14	14	
2	CST	358	346	M/64/L
	BCVA	20/60	20/30	
	IOP	16	12	
3	CST	463	321	F/80/R
	BCVA	20/60	20/40	
	IOP	12	12	
4	CST	406	334	M/72/R
	BCVA	20/400	20/160	
	IOP	12	12	
5	CST	342	291	M/82/L
	BCVA	20/70	20/50	
	IOP	14	12	
6	CST	613	409	F/74/L
	BCVA	20/400	20/160	
	IOP	12	16	
7	CST	389	257	M/75/R
	BCVA	20/100	20/60	
	IOP	16	16	
8	CST	337	286	F/86/L
	BCVA	20/100	20/80	
	IOP	16	16	
9	CST	528	407	M/76/L
	BCVA	20/320	20/250	
	IOP	18	16	
10	CST	364	324	F/61/L
	BCVA	20/100	20/70	
	IOP	16	16	
11	CST	783	543	M/72/L
	BCVA	20/70	20/30	
	IOP	14	16	

*CST* central subfield thickness measured by optical coherence tomography, *BCVA* logMAR best-corrected vision acuity, *IOP* intraocular pressure, *F* female, *M* male, *R* right eye, *L* left eye

### Full-field ERG response

**Fig. 1:**
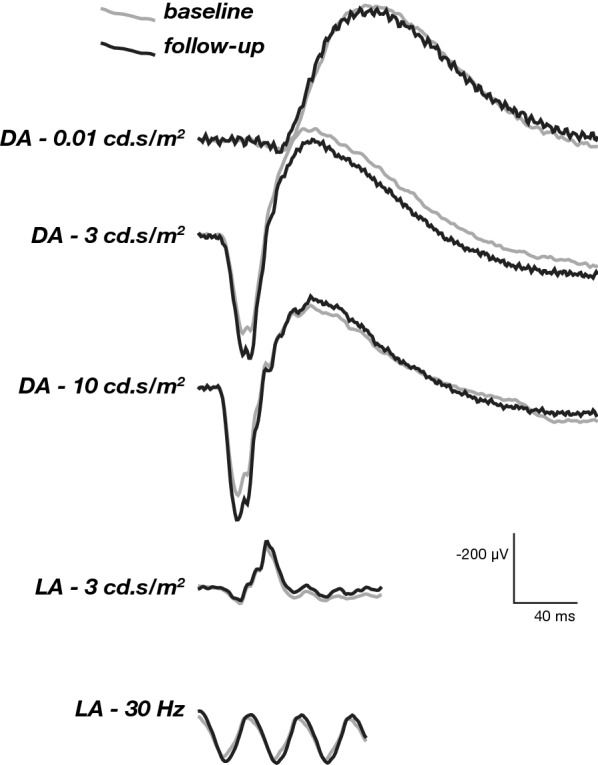
ERG changes. Full-field ERG baseline and after 12 weeks of follow up showed no significant difference between before and after treatment

There was no significant difference in the a- and b-wave amplitudes and implicit times for the dark-adapted ROD, COMBINED, and OSCILLATORY POTENTIAL responses and for the light-adapted CONE and FLICKER 30 Hz responses measured at baseline compared to 12 weeks (Table 2, Fig. 1).

**Table 2:** Dark and light-adapted ERG responses at baseline and 12-week study visits

ERG stimulus	Measurement	Baseline	Follow-up	P (Paired t-test)
DA—0.01	b-wave amplitude (µV)	195.6 ± 37.0	214.6 ± 28.9	0.5313
	b-wave implicit time (ms)	101.1 ± 3.7	102.1 ± 3.6	0.8158
DA—3.0	a-wave implicit time (ms)	23.2 ± 0.8	23.1 ± 0.8	0.9052
	a-wave amplitude (µV)	148.3 ± 23.2	193.2 ± 23.7	0.1840
	b-wave implicit time (ms)	65.3 ± 7.0	56.5 ± 1.4	0.2549
	b-wave amplitude (µV)	371.6 ± 44.2	425.9 ± 36.5	0.1840
	OP AUC (µV.ms)	483.9 ± 49.7	523.2 ± 117.5	0.5243
DA—10.0	a-wave implicit time (ms)	17.7 ± 1.0	18.0 ± 0.9	0.7324
	a-wave amplitude (µV)	199.8 ± 27.8	233.9 ± 24.0	0.1511
	b-wave implicit time (ms)	57.1 ± 1.8	60.5 ± 2.3	0.2353
	b-wave amplitude (µV)	377.9 ± 47.4	457.3 ± 34.2	0.0667
LA—30 Hz	Latency (ms)	39.6 ± 6.9	47.3 ± 6.9	0.2107
	Amplitude (µV)	89.2 ± 11.5	91.6 ± 9.1	0.7740
LA—3.0	a-wave implicit time (ms)	17.4 ± 0.3	18.0 ± 0.4	0.1107
	a-wave amplitude (µV)	27.1 ± 3.7	28.8 ± 1.5	0.6047
	b-wave implicit time (ms)	34.0 ± 0.5	34.4 ± 0.6	0.3966
	b-wave amplitude (µV)	107.6 ± 15.6	111.5 ± 9.1	0.6936

DA—0.01. Dark-adapted 0.01 ERG (a rod-driven ON bipolar cells response). DA—3.0. Dark-adapted 3 ERG (combined responses from photoreceptors and bipolar cells from rod and cone systems; rod dominated). Dark-adapted oscillatory potentials area under curve (OP AUC) (primarily from amacrine cells). DA—10.0. Dark-adapted 10 ERG (combined response with enhanced a-waves reflecting photoreceptor function). LA—30 Hz. Light-adapted 30 Hz flicker ERG (cone-pathway-driven response). LA—3.0. Light-adapted 3 ERG (a-waves from cone photoreceptors and cone OFF- bipolar cells; the b-wave are from ON- and OFF-cone bipolar cells)

### Intraocular pressure

**Fig. 2:**
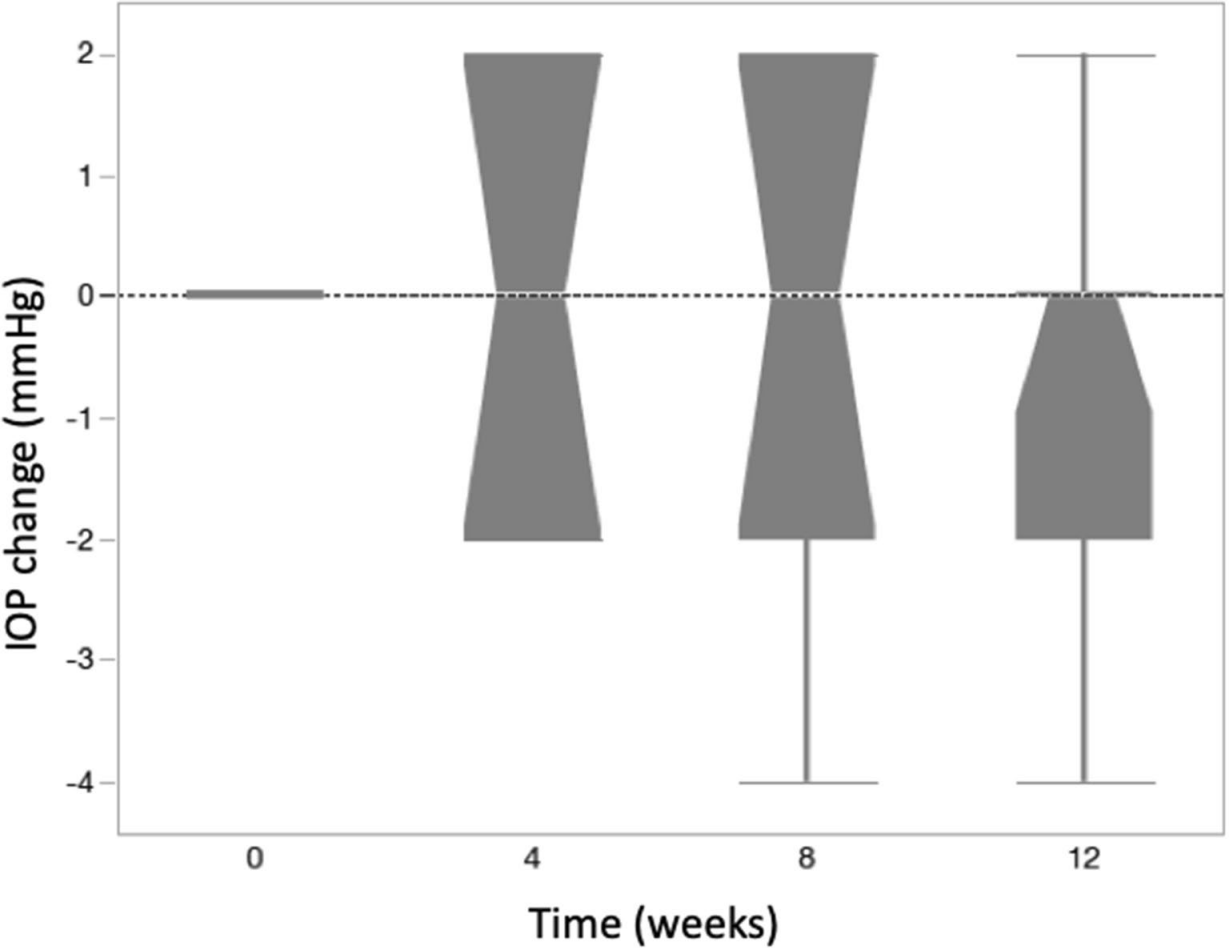
IOP (mmHg) changes at weeks 4,8 and 12. There was no significant change in mean IOP throughout the study period

The mean ± SE (Standard Error) IOP (mmHg) was 14.5 ± 0.6 at baseline; 14.3 mmHg ± 0.7 at week 4 (p = 0.99); 13.8 mmHg ± 0.5 at week 8 (p = 0.64); and 14.3 mmHg ± 0.5 at week 12 (p = 0.64). There was no significant change in mean IOP throughout the study period and none of the patients needed IOP-lowering eye drops or surgery (Fig. 2). Anterior chamber paracentesis was performed in 2 of the 11 patients because central retinal artery pulsation was identified under indirect ophthalmoscopy immediately after intravitreal injection.

### Best-corrected visual acuity (BCVA)

**Figure. 3:**
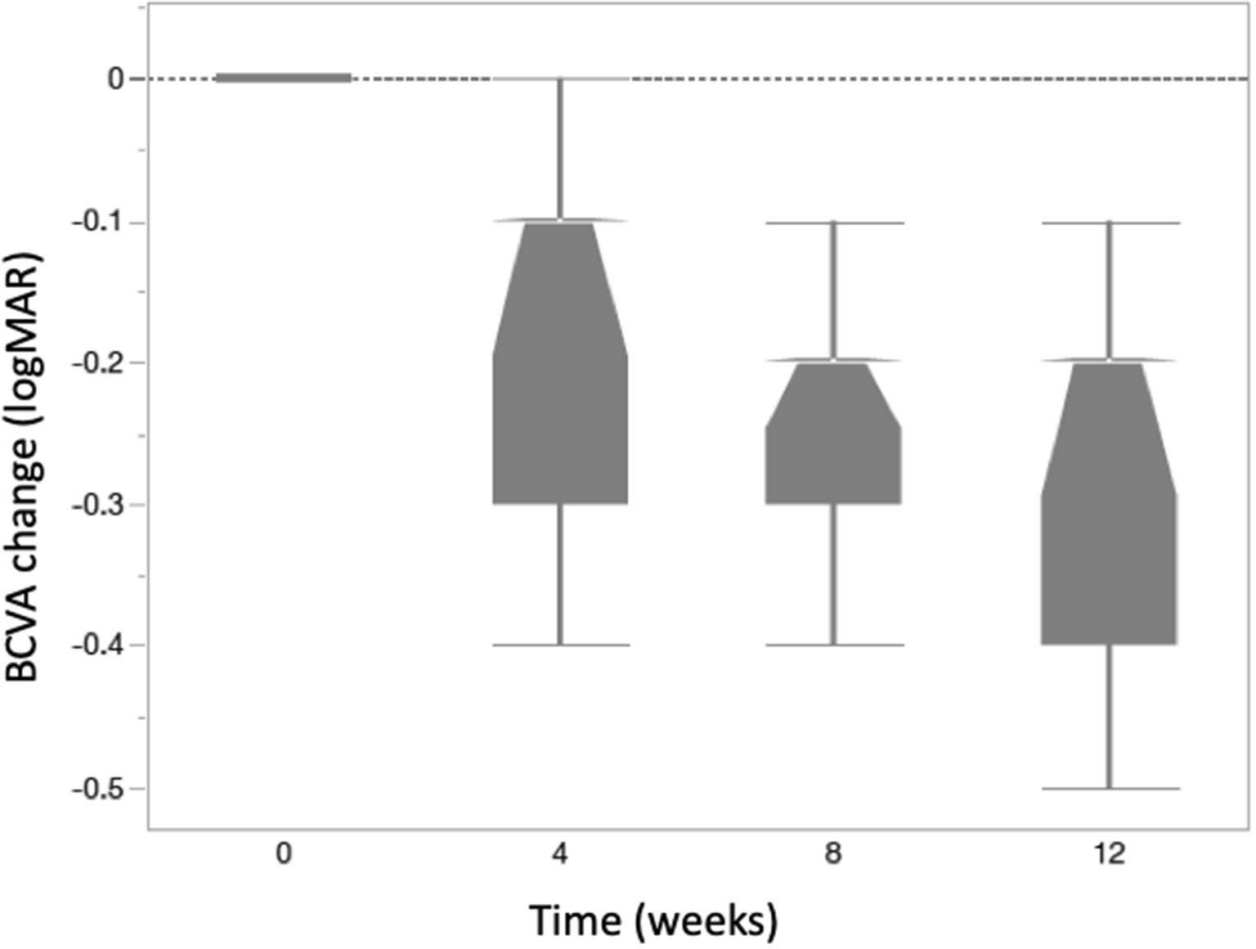
LogMAR BCVA changes at weeks 4, 8 and 12. Mean (± SE) logMAR BCVA improved by 0.173 ± 0.033 (p < 0.0001), 0.254 ± 0.033 (p < 0.0001) and 0.272 ± 0.033 (p < 0.0001) compared to baseline at 4, 8, and 12 weeks, respectively

At baseline, the mean ± SE logMAR BCVA was 0.79 ± 0.09. There was significant BCVA improvement compared to baseline at all study follow-up visits: mean ± SE logMAR BCVA was 0.61 ± 0.10 at week 4; 0.53 ± 0.10 at week 8; and 0.51 ± 0.09 at week 12. Mean (± SE) logMAR BCVA improved by 0.173 ± 0.033 (p < 0.0001), 0.254 ± 0.033 (p < 0.0001) and 0.272 ± 0.033 (p < 0.0001) compared to baseline at 4, 8, and 12 weeks, respectively (Figs. 3).

### Central subfield thickness (CST)

**Fig. 4:**
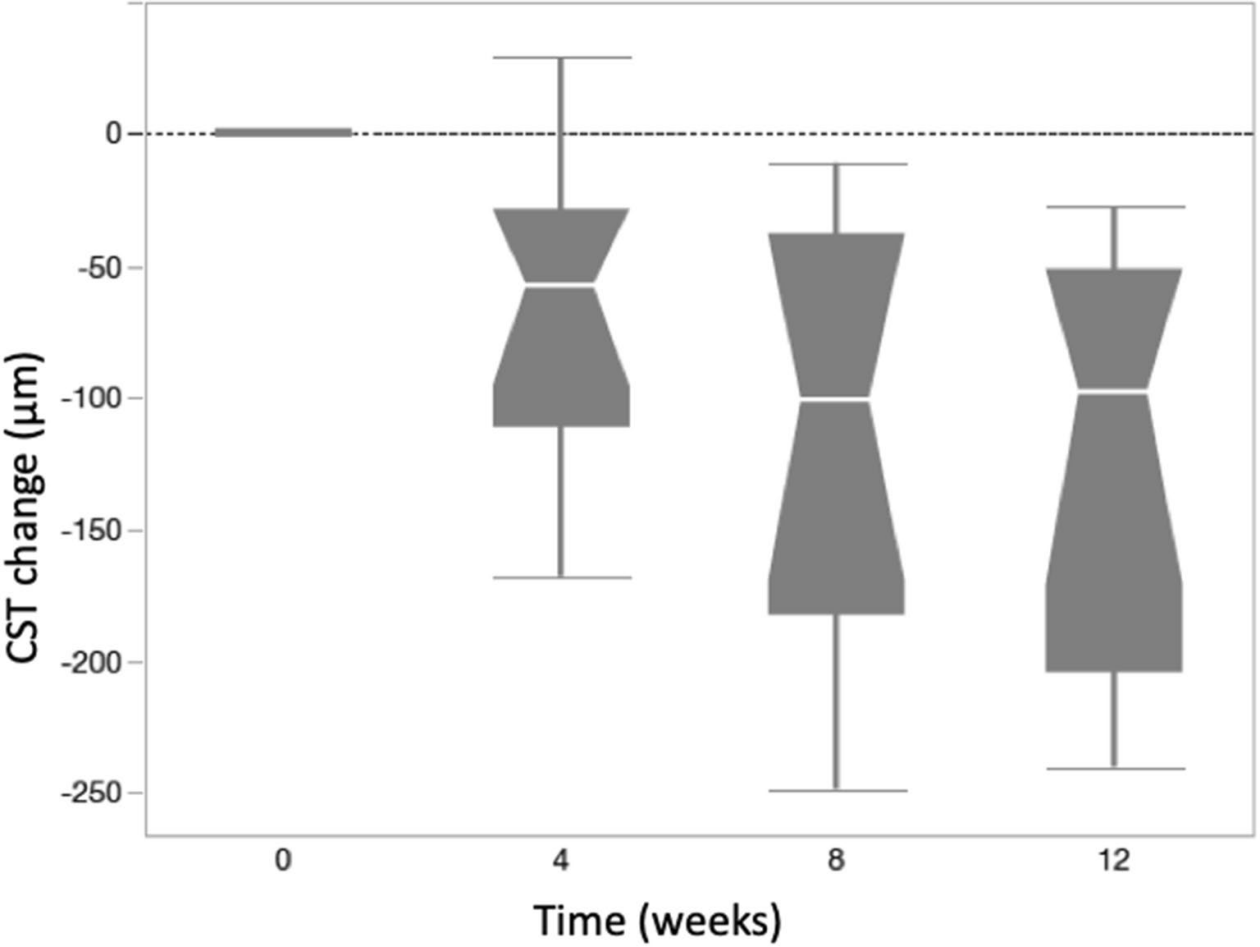
CST (μm) changes at weeks 4, 8 and 12. Mean (± SE) CST decreased from baseline by 77.64 ± 20.65 (p = 0.0039), 106.54 ± 20.65 (p < 0.0001) and 121.73 ± 20.65 (p < 0.0001) at 4, 8, and 12 weeks, respectively

**Fig. 5:**
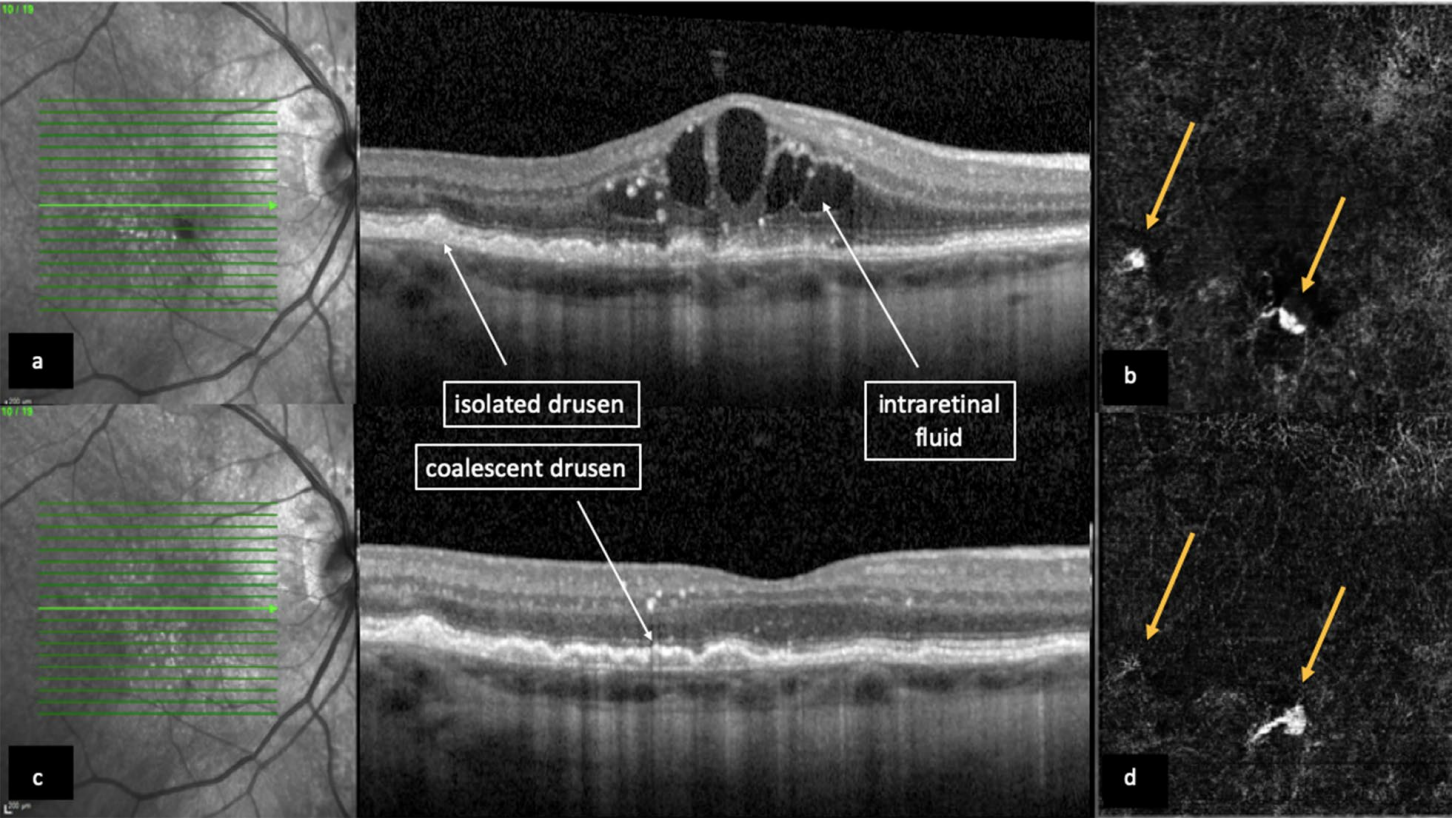
Spectral-domain OCT B-scan and angiography c-scan pictures from patient number 1. **a**. B-scan OCT at baseline showing isolated and coalescent drusen, junctional subretinal hyperreflective material, and intraretinal fluid. **b**. OCT-A picture showing two hyperreflective spots (yellow arrows) in the avascular complex slab, suggestive of choroidal neovascularization sites or foci. **c**. B-scan OCT at 12 week follow-up visit showing regression of intraretinal fluid and some junctional subretinal hyperreflective material. **d**. OCT-A picture showing discrete reduction of hyperreflectivity in temporal inferior CNV, while there was maintenance of flow in the inferior CNV focus

At baseline, the mean ± SE CST (μm) was 462 ± 45. There was significant CST reduction compared to baseline at all study follow-up visits: mean ± SE CST was 385 ± 37 at week 4; 356 ± 29 at week 8 and 341 ± 24 at week 12. Mean (± SE) CST decreased from baseline by 77.64 ± 20.65 (p = 0.0039), 106.54 ± 20.65 (p < 0.0001) and 121.73 ± 20.65 (p < 0.0001) at 4, 8, and 12 weeks, respectively (Figs. 4, 5).

### Other adverse advents

During the 12 week follow-up period, none of the study eyes were observed to have intraocular inflammation, endophthalmitis or an increase in lens opacity.

## Discussion

To our knowledge, and based on a computerized search of the Medline database, the current study is the first to assess the in vivo safety of combined intravitreal bevacizumab and propranolol to treat an ocular neovascular disease. The disease selected for study was nARMD due to its prevalence and worldwide public health impact. Due to the neovascular pathophysiology of this disease, we hypothesized that the combined use of two drugs with known anti-VEGF properties may have synergistic effects and, if safe, the addition of the inexpensive propranolol medication could reduce the need for frequent retreatments with expensive intravitreal anti-VEGF agents. This phase 1 trial was focused on the safety of the combination therapy.

The propranolol dosage of 50 µg was selected based on a report by Karimi et al. [15] who treated a patient with retinal capillary hemangioma with intravitreal propranolol injections. In their study, as in the present one, there were no significant changes on ERG waves amplitudes after treatment. Another previous study used intravitreal propranolol injections in an animal model (rabbits) [25]. In addition, studies used another b-blocker, metoprolol, intravitreally and reported that no evidence of retinal toxicity was observed in rabbits [26] and humans [27–29].

Bevacizumab and propranolol have already been used as solutions in the same dosage as the ones employed in the current study [15, 30]. Regarding a possible negative interaction between drugs, there is a previous report of synergistic effects of propranolol and bevacizumab, in vitro, to inhibit the growth of human umbilical vein endothelial cells (HUVECs) and BJ human normal fibroblasts (BJs) [31].

In addition to the absence of ERG changes identified in the current study, no anterior chamber cells or flare were observed following the use of combination bevacizumab and propranolol injections. These findings are consistent with other studies in which intravitreal b-blockers were studied [15, 27–29].

There was no significant increase in mean IOP during the study. Since 0.1 ml intravitreal injections may cause an acute IOP peak, we tried to avoid that by using an oral dose of 250 mg acetazolamide 30 minutes prior to each injection, unless medically contraindicated, and we performed indirect ophthalmoscopy immediately after the injection to check the perfusion of the optic nerve. Despite these procedures, two patients were treated with anterior chamber paracentesis due to central retinal artery pulsation verified under indirect ophthalmoscopy. It is important to consider previous glaucoma diagnosis in patients being evaluated for 0.1 ml injections. Fortunately, none of the enrolled patients had a previous diagnosis of glaucoma, an IOP higher than 21 mmhg at baseline examination, or an optic disc vertical and horizontal cup-to-disc ratio larger than 0.5.

Best-corrected visual acuity results also support the safety of the combined intravitreal bevacizumab and propranolol investigated in the present study, since there was significant BCVA improvement compared to baseline at all study follow-up visits. The BCVA improvement at week 12 was 0.272 ± 0.033 (p < 0.0001), which corresponds to an improvement of 13.6 ETDRS letters. Rich et al. [32] used bevacizumab monotherapy for patients with nARMD, with a similar follow-up period of 3 months; injections were performed at 4 week intervals, as needed, based on persistence of retinal fluid, and the BCVA improvement at 3 months compared to baseline was 7.9 letters, (approximately 0.15 log MAR BCVA), which is lower than what we observed in our study. Also, patients in our study had a mean CST reduction of 121.73 μm when compared to baseline, while in the study by Rich et al. [32] the mean total decrease in CST at 3 months compared to baseline was 99.6 μm. The better outcomes in our study could be due to the combination of the drugs, or may be related to a difference in anti-VEGF treatment regimen. Rich et al. used a 4 week interval as needed (43% needed 3–4 injections, 36% 2 injections, and a total of 79% of eyes received retreatment) and our study used a fixed 4 week interval regimen (for a total of 3 injections).

## Conclusions

The current study is the first to evaluate retinal toxicity following administration of combination bevacizumab and propranolol injections using both functional and structural tests in humans. Study limitations include a short follow-up period, lack of a control group, and absence of other functional tests such as microperimetry and contrast sensitivity measurements.

In summary, monthly 0.1 ml intravitreal injections of a combination of bevacizumab (1.25 mg/0.05 ml) and propranolol (50 g/0.05 ml) during a 12 week period appears to be safe and cause no signs of acute toxicity. Further studies with a larger number of patients, longer follow-up, and control group (bevacizumab monotherapy) are warranted to assess the potential value of this combined alternative therapy.

## Data Availability

The data generated or analyzed during this study are included in supplementary information files. Any other information/data are available from the corresponding author on reasonable request.

## Abbreviations

nARMD: Neovascular age-related macular degeneration
RPE: Retinal pigment epitelium
VEGF: Anti-vascular endothelial growth factor
b-AR: Beta-adrenergic receptor
OIR: Oxygen-induced ischemic retinopathy
CNV: Choroidal neovascularization
BCVA: Best-corrected visual acuity
ETDRS: Early treatment diabetic retinopathy study
ISCEV: International society for clinical electrophysiology of vision
OCT: Optical coherence tomography
OCT-A: OCT angiography
ERG: Electroretinography
CST: Central subfield thickness
IOP: Intraocular pressure
MANOVA: Multiple analysis of variance
HUVEC: Human umbilical vein endothelial cells
